# Associations Between Personality Traits and Energy Balance Behaviors in Emerging Adulthood: Cross-Sectional Study

**DOI:** 10.2196/42244

**Published:** 2023-06-15

**Authors:** Katrina E Champion, Cath Chapman, Matthew Sunderland, Tim Slade, Emma Barrett, Erin Kelly, Lexine Stapinski, Lauren A Gardner, Maree Teesson, Nicola C Newton

**Affiliations:** 1 The Matilda Centre for Research in Mental Health and Substance Use The University of Sydney Sydney Australia

**Keywords:** personality, emerging adulthood, screen time, sedentary, sleep, diet, physical activity, prevention, mental health, risk factor, sedentary behavior, chronic disease

## Abstract

**Background:**

Internalizing and externalizing personality traits are robust risk factors for substance use and mental health, and personality-targeted interventions are effective in preventing substance use and mental health problems in youth. However, there is limited evidence for how personality relates to other lifestyle risk factors, such as energy balance–related behaviors, and how this might inform prevention efforts.

**Objective:**

This study aimed to examine concurrent cross-sectional associations between personality traits (ie, hopelessness, anxiety sensitivity, impulsivity, and sensation seeking) and sleep, diet, physical activity (PA), and sedentary behaviors (SB), 4 of the leading risk factors for chronic disease, among emerging adults.

**Methods:**

Data were drawn from a cohort of young Australians who completed a web-based, self-report survey in 2019 during early adulthood. A series of Poisson and logistic regressions were conducted to examine the concurrent associations between the risk behaviors (sleep, diet, PA, and sitting and screen time) and personality traits (hopelessness, anxiety sensitivity, impulsivity, and sensation seeking) among emerging adults in Australia.

**Results:**

A total of 978 participants (mean age 20.4, SD 0.5 years) completed the web-based survey. The results indicated that higher scores on hopelessness were associated with a greater daily screen (risk ratio [RR] 1.12, 95% CI 1.10-1.15) and sitting time (RR 1.05, 95% CI 1.0-1.08). Similarly, higher scores on anxiety sensitivity were associated with a greater screen (RR 1.04, 95% CI 1.02-1.07) and sitting time (RR 1.04, 95% CI 1.02-1.07). Higher impulsivity was associated with greater PA (RR 1.14, 95% CI 1.08-1.21) and screen time (RR 1.06, 95% CI 1.03-1.08). Finally, higher scores on sensation seeking were associated with greater PA (RR 1.08, 95% CI 1.02-1.14) and lower screen time (RR 0.96, 95% CI 0.94-0.99).

**Conclusions:**

The results suggest that personality should be considered when designing preventive interventions for lifestyle risk behaviors, particularly in relation to SB, such as sitting and screen time.

**Trial Registration:**

Australian New Zealand Clinical Trials Registry ACTRN12612000026820; https://tinyurl.com/ykwcxspr

## Introduction

Chronic diseases, such as cancer, cardiovascular disease, and mental disorders, are the leading causes of disability and death across the globe [1]. It is estimated that almost one-third of chronic diseases could be prevented by reducing exposure to modifiable lifestyle risk behaviors [2], such as physical inactivity, poor diet, alcohol use and smoking, poor sleep, and sedentary behaviors (SB; sitting and screen time) [3,4]. These risk behaviors are highly prevalent among adolescents in Australia [5] and worldwide [6] and are associated with significant health consequences, including increased risk of obesity [7] and emotional and behavioral problems [8,9]. They typically have their onset early in life, and once established, they track into adulthood, increasing the risk for chronic disease over the life course [4,10-12]. Identifying and implementing innovative strategies to prevent or modify these lifestyle risk behaviors during adolescence is critical for promoting lifelong health.

One plausible target for preventing lifestyle risk behaviors and associated harms is personality traits. Numerous studies have demonstrated robust associations between personality traits and substance use [13], particularly with respect to the internalizing personality profiles of *anxiety sensitivity* (fear of anxiety-related physical sensations) and *hopelessness* (tendency toward low mood; worthlessness; and negative beliefs about oneself, the world, and the future), and the externalizing profiles of *sensation seeking* (elevated need for stimulation and intolerance to boredom) and *impulsivity* (rapid decision-making and poor response inhibition) [14]. Each personality trait has been shown to be associated with specific patterns of substance misuse, motivations for use, and comorbid psychopathology. For example, hopelessness has been associated with the early onset of alcohol use and depressive symptoms, while impulsivity has been associated with drinking problems, tobacco use, other drug use, and conduct problems [15]. These personality profiles have been shown to be reliable predictors of future substance use and mental health problems and are therefore important targets for screening and prevention [13,15-17].

Improved understanding of the way in which personality traits are associated with other highly prevalent, yet modifiable risk behaviors among youth has considerable implications for tailoring prevention programs to account for particular personality styles. To date, much of the research examining associations between personality and energy balance–related behaviors (ie, health behaviors related to energy intake and energy expenditure) such as dietary intake, SBs (sitting and screen time), physical activity (PA), and sleep has focused on children and early adolescents [18,19]. For example, a 2019 cross-sectional study among 8-11 year olds in the United States found that adequate sleep duration and reduced screen time were associated with less impulsive behavior among children [18]. On the contrary, there is little research on the associations between personality and lifestyle risk behaviors during emerging adulthood (18-25 years), a critical, yet poorly understood period characterized by numerous personal and social role changes, increased autonomy over lifestyle choices, declines in health behaviors [20,21], and increased risk of anxiety, depression, and substance use disorders [22]. The limited existing literature on this population has found relationships between sleepiness and impulsivity [23] and extraversion and SB [24]. However, further research among larger samples of emerging adults and addressing a broader range of energy balance–related risk behaviors is required. Therefore, this study aimed to examine cross-sectional associations between 4 personality traits (hopelessness, anxiety sensitivity, impulsivity, and sensation seeking) and 5 energy balance–related behaviors (screen time, sitting time, fruit and vegetable intake, sleep duration, and moderate-to-vigorous physical activity [MVPA]) among a sample of emerging Australian adults.

## Methods

### Study Design

The sample was derived from the long-term follow-up of an existing cohort of young Australians who participated in a cluster randomized controlled trial (RCT). A total of 2190 grade 8 students (54% male; mean age 13.3, range 11.9-15.4 years) from 26 Australian secondary schools in New South Wales and Victoria (17 private or independent and 9 public) were originally recruited to the RCT in 2012. This 4-arm cluster RCT evaluated the impact of universal and selective interventions (separately and together) for the prevention of alcohol and other drug use delivered in grade 8. Intervention content did not focus on the behaviors measured for this study. All participants had received health education as usual (either as part of the control arm of the study in grade 8) or over the period of follow-up in grades 9 and 10 at school. This trial is registered with the Australian New Zealand Clinical Trials Registry (ACTRN12612000026820), and full details of the RCT, including detailed descriptions of the interventions, adherence, and attrition are published elsewhere [25-27]. Despite the longitudinal nature of the cohort, given data on healthy lifestyle behaviors were only obtained during the final follow-up period, this study uses cross-sectional data collected from 978 participants (mean age 20.4, SD 0.5 years) who completed the 7-year follow-up assessment in 2019.

### Procedure

Using multiple sources of locator information provided during previous assessments (including email, phone number, postal address, Facebook handle, and parents’ email address), participants were contacted and invited to complete a web-based, self-report survey at a time and location of their choice.

### Ethics Approval

All procedures were approved by the Human Research Ethics Committees of the University of New South Wales (HC16881) and The University of Sydney (HREC 2018/845). All participants were required to provide digital informed consent prior to participation, which allows secondary analyses, such as this study, without additional consent. To ensure privacy and confidentiality, all data were deidentified. Respondents were compensated AUD $30 (USD $19.80) (or an equivalent voucher) for their time.

### Measures

#### Overview

All measures were self-report and were completed digitally.

#### Demographics

The following demographic variables were assessed: sex (male, female, and other), employment status (full-time, part-time, unemployed, and other), and tertiary education in progress or completed (none, trade/technical, and university/college).

#### Personality

Personality was measured using the Substance Use Risk Profile Scale (SURPS), a 23-item tool developed to assess 4 personality traits relevant to substance use: impulsivity, hopelessness, anxiety sensitivity, and sensation seeking [13,16]. The tool measures 4 dimensions of personality: impulsivity (eg, I often don’t think things through before I speak), sensation seeking (eg, I enjoy new and exciting experiences even if they are unconventional), hopelessness (eg, I feel that I am a failure), and anxiety sensitivity (eg, I get scared when I experience unusual bodily sensations). The SURPS is scored on a 4-point Likert scale (strongly agree—strongly disagree) and has demonstrated acceptable to good internal consistency and reliability (α=.62-.86) as well as good concurrent and predictive validity among a comparable sample of Australian youth [15]. The total scores for each of the 4 subscales were calculated and converted into *z*-scores based on the total sample mean and SD.

#### Fruit and Vegetable Consumption

Fruit and vegetable intake were assessed using 2 validated items commonly used in health research [28,29]: “About how many serves of fruit/vegetables do you usually have each day?” Possible response options were “don’t eat fruit/vegetables,” “1 serve or less,” “2-3 serves,” “4-5 serves,” and “6 serves or more.” Participants were provided with written information about what constitutes one serving of fruit and vegetables. In line with the Australian dietary guidelines [30], responses were dichotomized so that poor fruit intake was defined as less than 2 servings per day, and insufficient vegetable intake was classified as less than 4-5 servings per day. A new variable was constructed to represent both fruit and vegetable intake with two categories: (1) adequate fruit or vegetable intake and (2) inadequate fruit and vegetable intake.

#### Physical Activity

Self-reported MVPA was assessed using items from the International Physical Activity Questionnaire-Short Form (IPAQ-SF), which has demonstrated good psychometric properties in a diverse range of samples [31]. Respondents were asked to indicate how many days during the past 7 days and for how long each day (in hours and minutes) they had performed vigorous PAs (eg, heavy lifting, digging, aerobics, or fast bicycling)*.* Participants were also asked to report the number of days they did moderate PAs (eg, carrying light loads, bicycling at a regular pace, or doubles tennis, excluding walking) in the past 7 days and how much time they usually spent doing these activities on one of those days (in hours and minutes). The number of hours spent performing both vigorous and moderate PA in the past 7 days was summed and divided by 7 to calculate the average number of hours per day engaged in MVPA.

#### Sedentary Behaviors

Self-reported daily sitting time (in hours) was assessed using a single item from the IPAQ-SF [31]:

How many hours do you spend sitting in a typical 24-hour day (eg, travelling to/from school, university or work; at school, university or work; watching television, using a computer at home and leisure time).

To assess screen time, respondents were asked to report the amount of time (hours and minutes) in the past week they typically spent (1) watching television or videos during their free time and (2) using a computer during their free time (including computers, laptops, Xbox, PlayStation, iPads or other tablets, smartphones, YouTube, Facebook or other social media, and the internet). Separate items were used to assess average screen time on weekdays and weekend days. The total number of hours per week spent on both TV and computer during their free time was calculated by multiplying the sum of weekday TV and computer time by 5, multiplying the sum of weekend day TV and computer time by 2, and dividing by 7 to generate an average screen time per day over a typical week.

#### Sleep Duration

To assess sleep duration (in hours), respondents were asked “How many hours in each 24-hour day do you usually spend sleeping (including at night and naps)?” The total number of hours was used as a continuous indicator of sleep.

### Statistical Analyses

Given prior work has demonstrated sex differences in the prevalence of lifestyle risk behaviors [32,33], descriptive statistics (means and frequencies) were determined for each of the risk behaviors by sex. Inspection of the outcomes in terms of the underlying distribution were used to inform the selection of appropriate regression models. A series of Poisson (for MVPA, sleep, screen time, sitting time) with a log link, and logistic regressions (for fruit or vegetable intake) with a logit link were estimated to examine the associations between risk behaviors and personality type. Rate ratios (RRs) derived by exponentiating the coefficients from Poisson regressions were presented for semicontinuous count data (MVPA, sleep, screen time, and sitting time), whereas odds ratios (ORs) derived by exponentiating the coefficients from logistic regressions were presented for binary data (fruit or vegetable intake). Initial data inspection indicated that the semicontinuous count variables (MVPA, sleep, screen time, and sitting time) demonstrated a significant negative skew, indicative of count data and suitable for Poisson models in comparison to linear models. Separate models were estimated to examine the association of each personality type with the multiple health behavior outcomes with sex, education status, and employment included in each model as covariates. Additional sensitivity analyses were conducted by reestimating the Poisson and logistic regression models for each outcome but included all personality scores in a single model as well as the additional covariates of sex, education status, and employment to determine independent effects of personality scores on each risk behavior. All analyses were conducted in SAS (version 9.4; SAS Institute).

## Results

### Sample Characteristics

A total of 978 participants (n=478, 49% male; mean age 20.4, SD 0.4 years) completed the web-based survey. Table 1 summarizes the sample characteristics, and Table 2 reports descriptive statistics for the lifestyle risk behaviors by sex among those who provided adequate data for the risk behaviors. Overall, the means and SDs of the raw personality scores are 13.6 (4.1) for negative thinking, 12.0 (2.9) for anxiety sensitivity, 10.0 (2.7) for impulsivity, and 15.7 (3.8) for sensation seeking.

**Table 1:** Sociodemographic characteristics of the sample at the 2019 assessment wave.

Characteristic		Values
**Age (years)**		
	Mean (SD)	20.4 (0.4)
	Range	18.2-22.0
**Sex, n (%)**		
	Male	478 (48.9)
	Female	499 (51.1)
**Tertiary education (completed or in progress), n (%)**		
	None	36 (4)
	Trade or technical	88 (9)
	University or college	854 (87.3)
**Employment status, n (%)**		
	Full-time	100 (10.2)
	Part-time	707 (72.3)
	Unemployed	157 (16.1)
	Other	14 (1)

**Table 2:** Prevalence of lifestyle risk behaviors by sex at the 2019 assessment wave.

	MVPA^a^ (n=829; h/day), mean (SD)	Sleep duration, (n=977; h/day), mean (SD)	Screen time (n=880; h/day), mean (SD)	Sitting time (n=974; h/day), mean (SD)	Fruit or vegetable (n=977), % inadequate intake/day^b^
Total sample	1.6 (2.7)	7.9 (2.4)	7.7 (6.1)	7.4 (3.4)	84.4
Male	2.0 (2.6)	7.8 (2.2)	8.1 (6.7)	7.3 (3.4)	89.3
Female	1.2 (2.7)	7.9 (2.5)	7.3 (5.4)	7.5 (3.3)	79.8

^a^MVPA: moderate-to-vigorous physical activity.

^b^Combines participants with inadequate fruit or vegetable intake.

### Associations Between Personality and Lifestyle Risk Behaviors

Table 3 presents the RR, OR, and 95% CI from the regression models. Results indicated that a 1 SD higher score on hopelessness was associated with a 12% higher rate of screen time (RR 1.12, 95% CI 1.10-1.15) and a 5% higher rate of sitting time (RR 1.05, 95% CI 1.03-1.08). Similarly, a 1 SD higher score on anxiety sensitivity was associated with a 4% higher rate of both screen time and sitting time (RR 1.04, 95% CI 1.02-1.07; RR 1.04, 95% CI 1.02-1.07). A 1 SD higher score on impulsivity was associated with a 14% higher rate of MVPA (RR 1.14, 95% CI 1.08-1.21) and a 6% higher rate of screen time (RR 1.06, 95% CI 1.03-1.08). Finally, a 1 SD higher score on sensation seeking was associated with an 8% higher rate of MVPA (RR 1.08, 95% CI 1.02-1.14) and a 4% lower rate of screen time (RR 0.96, 95% CI 0.94-0.99). Personality was not associated with inadequate fruit and vegetable intake in any of the models (RRs 0.88-1.18; all CIs included 1.0). Sensitivity analyses adjusted for all other personality scores in the models are provided in Multimedia Appendix 1 and demonstrate the robustness of the key findings.

**Table 3:** Rate ratios, odds ratios, and 95% CI from the regression models examining associations between personality type and lifestyle risk behaviors at the 2019 assessment wave.

Personality type	MVPA^a^ (n=829), RR^b^ (95% CI)	Sleep duration (n=977), RR (95% CI)	RR (95% CI)	Sitting time (n=974), RR (95% CI)	Inadequate fruit or vegetable^c^ (n=977), OR^d^ (95% CI)
Hopelessness	0.95 (0.90-1.01)	1.00 (0.98-1.02)	*1.12 (1.10-1.15)^e^*	*1.05 (1.03-1.08)*	1.18 (0.99-1.41)
Anxiety sensitivity	1.02 (0.97-1.08)	1.01 (0.99-1.03)	*1.04 (1.02-1.07)*	*1.04 (1.02-1.07)*	0.91 (0.76-1.09)
Impulsivity	*1.14 (1.08-1.21)*	0.99 (0.97-1.01)	*1.06 (1.03-1.08)*	0.99 (0.96-1.01)	1.05 (0.87-1.25)
Sensation seeking	*1.08 (1.02-1.14)*	0.99 (0.97-1.02)	*0.96 (0.94-0.99)*	0.98 (0.96-1.00)	0.88 (0.74-1.06)

^a^MVPA: moderate-to-vigorous physical activity.

^b^RR: risk ratio.

^c^Odds ratios derived from logistic regression with reference category as adequate fruit and vegetable intake. All models controlled for sex, employment status, and education.

^d^OR: odds ratio.

^e^Italics indicates significance at the *P*<.05 level.

## Discussion

### Principal Results

This study examined the cross-sectional associations between personality traits (hopelessness, anxiety sensitivity, sensation seeking, and impulsivity) and sleep duration, diet, PA, and SB, 4 of the leading risk factors for chronic disease among a sample of emerging Australian adults. We found that hopelessness was associated with greater screen and sitting time, and anxiety sensitivity was associated with higher screen and sitting time. In terms of the externalizing personality traits, impulsivity was associated with greater screen time and MVPA, while sensation seeking was associated with greater MVPA but less screen time. There were no significant associations between any of the personality traits and sleep or diet.

### Comparison With Prior Work

This study adds to the limited literature on personality traits and energy balance–related behaviors during emerging adulthood. The finding that participants high on hopelessness engaged in greater daily screen and sitting time among our sample of Australian 20 year olds is consistent with prior research among young people. For example, a longitudinal study conducted among a community-based sample of Canadian youth (spanning ages 10-21 years) similarly found that higher levels of depression were associated with greater screen time [34]. Although the personality trait of hopelessness measured in our study is not a direct measure of depressive symptomology, previous research has shown that the 2 constructs are moderately to strongly correlated [15,35]. The finding is also consistent with coping styles associated with hopelessness (and depressive symptomatology) such as withdrawal from others, avoidance of in-person social interaction, and distraction [13]. Relatedly, the present findings support previous work that has found the symptoms of depression to be associated with reduced PA among both adolescents and young adults [34,36], likely reflective of the Diagnostic and Statistical Manual of Mental Disorders (DSM-5) Diagnostic Criteria for depression of reduced physical movement and loss of energy. Our findings that impulsivity was associated with greater screen time, and sensation seeking with less screen time, is largely consistent with prior work [18]. For example, a small study among young Australian adults found that extraversion, which is correlated with the SURPS sensation-seeking scale [16], was negatively associated with both sitting time and leisure screen time [24], and a study among Spanish adolescents showed that extraversion was linked to a reduced risk of excessive use of social network sites [19]. Taken together, it seems that although young people exhibiting high levels of sensation seeking may have an increased risk of substance use and mental health problems [15], they are less likely to engage in risky levels of screen time and physical inactivity. Those exhibiting hopelessness and anxiety sensitivity, on the other hand, are more likely to engage in risky levels of screen time and sitting time as well as being at risk of substance use and mental health problems [15].

Interestingly, we did not find evidence of an association between the 4 personality traits and inadequate diet or less sleep. The lack of an association between personality and dietary intake is likely due to the fact that only fruit and vegetable consumption was measured, and we did not assess other dietary behaviors such as intake of sugar-sweetened beverages or junk food. Indeed, prior research suggests that individuals high on impulsivity are more likely to find it difficult to resist impulses related to unhealthy food choices [37], and impulsivity is associated with excessive overeating of high-calorie food or food addiction [38]. Similarly, our results in terms of sleep and personality somewhat contradict prior work that has found associations between sleep problems and impulsivity among young adults in the United States; however, the US-based study assessed daytime sleepiness, rather than total sleep duration [23]. It is also worth highlighting that self-reported sleep duration in the present sample was relatively high, with a mean of 7.9 (SD 2.4) hours of sleep reported per day. Although this is comparable to a recent Australian cohort where young adults (aged 18-24 years) reported sleeping 7.2 hours on average per night [39], it falls within the Australian health guidelines for sleep (7-9 hours per night) among young adults, so this may have limited our ability to examine associations between personality and sleep duration among those at the lower end of the sleep spectrum. Finally, our study did not assess sleep quality, which recent studies have suggested could be a better measure than sleep quantity as an index for assessing sleep, and further that in people with an average sleep duration of 7 hours, average sleep *quality* may be better related to health and other factors [40]. Future studies that include more nuanced sleep measures may shed more light on the relationship between personality traits and sleep. Similarly, levels of MVPA were also high among our sample. While a recent Australian study using self-reported data from the National Health Survey, estimated over half (55% or 1.2 million) of all 18-24 year olds were sufficiently active for their age [41], future studies with samples reporting MVPA levels at the lower end of the spectrum would be useful.

### Implications for Prevention

The findings from this study have important implications for the prevention of chronic disease risk factors, especially excessive screen time, a highly prevalent behavior that has increased among children and adults in the wake of the COVID-19 pandemic [42,43]. Indeed, in 2019, before the global pandemic, this sample of young adults reported very high rates of daily recreational screen time, with an average of 7.7 (SD 6.1) hours of screen time per day (including watching TV or videos, using a computer, or playing videogames). Excessive sitting time was also prevalent, with participants reporting sitting a mean of 7.4 (SD 3.4) hours per day. High levels of SB have been associated with a range of negative health consequences [44]. Among adolescents, for example, excessive screen time is linked to markers of adiposity and cardiometabolic disease risk [45], poor mental health [46] and quality of life [47], and increased risk of all-cause, cardiovascular disease, and cancer-related mortality, and incidence of these diseases, in adults [48,49]. Therefore, public health approaches to modify SB are critical. Prior research has suggested that understanding the ways in which personality and risk behaviors are related may be important for designing more effective and appealing public health campaigns [36]. For example, a campaign that links reductions in SB to changes in mood may be more appealing to young adults than a campaign that simply focuses on moving more.

In addition, our results suggest that identifying young people with high levels of hopelessness, anxiety sensitivity, and impulsivity could be a means of intervening to reduce SB, such as sitting and screen time, and increasing MVPA. For example, a program targeting young people with high hopelessness could also teach young people strategies to improve both their mood and physical health, such as reducing screen time, spending time with others outdoors, and engaging in PA. Designed to target young people based on personality risk factors for substance misuse and other emotional and behavioral problems, the *Preventure* intervention is a selective, personality-targeted program that has been shown to reduce alcohol use, alcohol-related harms, illicit substance use, depressive symptoms, delinquency, truancy, bullying, and conduct problems in RCTs in North America and Australia up to 7 years post intervention [26,50-53]. It is a brief, manual-based intervention that includes character profiles and case studies to teach young people about their target personality style, help them to explore ways of coping with unhelpful thoughts and emotions related to their personality, and to identify and challenge personality-specific cognitions that lead to problematic behaviors [13,54]. Adaptations to programs such as Preventure could include specific personality-tailored content aimed at lifestyle risk behaviors such as screen time and sleep, as well as concurrent targeting of several related lifestyle risk behaviors, potentially offering synergistic effects on multiple risk behaviors [55].

### Limitations

There are several limitations that should be considered. First, our measures of lifestyle risk behaviors were self-reported and were limited to simple indicators of each behavior. Future research should aim to assess a broader range of outcomes, including sugary foods and drinks, sleep quality, and specific types of screen time, including device and content viewed, and different types of PA (eg, active transport). Future research could also consider the inclusion of objective measures of lifestyle risk behaviors, noting that the benefits need to be weighed against the higher costs and practicalities of assessment as well as the potential barriers to engagement among young adults. Second, the SURPS differs from other widely used measures of personality, such as the Big 5 model of personality (neuroticism, extraversion, openness, agreeableness, and conscientiousness). Numerous studies have demonstrated the SURPS to be a reliable tool for identifying personality traits associated with substance misuse and other emotional and behavioral problems among adolescents [15-17,56]. Noting this, a key advantage of the SURPS is that its items measure personality traits and not substance use. This enables the identification of adolescents at greater risk for substance use and related risk behaviors prior to their entrenchment, which is particularly beneficial for the implementation of selective or targeted preventive interventions.

Third, the original sample was designed as a cluster RCT for a school-based prevention program on harmful alcohol use rather than a representative sample of Australian young adults, and covariates included in the analyses were limited to those measured in the original study. There were a large number of schools in the trial, and they represented both independent and public schools in 2 states in Australia. However, the sample was largely Australian-born, English-speaking adolescents, and although this is in line with the general population [57], they were primarily well educated and employed, limiting the generalizability of our findings. This is particularly important, given the prevalence of risk factors is not uniform across populations. For example, low socioeconomic status youth in Australia are 22 times more likely to use alcohol at risky levels and 5 times more likely to smoke [58], and 12% less likely to be sufficiently physically active [59] than higher socioeconomic status youth. Co-designed interventions for disadvantaged populations are clearly needed, including interventions delivered in the home and school settings as well as public health initiatives to address the underlying structural and social determinants of health. Future research examining the associations between personality and chronic disease risk factors among young adults from diverse and disadvantaged backgrounds is required to inform such interventions, along with the inclusion of a broader range of potential covariates related to socioeconomic status and disadvantage.

Fourth, we note that there is considerable attrition at the time point when lifestyle risk behaviors were obtained compared to the baseline sample (55%), and therefore, some degree of response bias might limit our results. Comprehensive attrition analyses are reported elsewhere [26]; however, those who were missing at time points 6 and 7 compared to those who were retained were more likely to be male, less likely to have used alcohol or have hazardous levels of drinking at baseline, and demonstrated lower mean impulsivity and sensation-seeking scores at baseline. Replication of these findings in other samples would support the robustness of the findings reported here. Finally, this analysis was limited to cross-sectional examinations, as the lifestyle risk behaviors (ie, diet, PA, and SB) were only assessed at the early adulthood assessments of the RCT, limiting our ability to examine causal and bidirectional relationships between personality and lifestyle risk behaviors. Nonetheless, this study focuses on the critical and understudied emerging adulthood period and suggests that young adults exhibiting high levels of specific personality traits may benefit from targeted interventions to improve both physical and mental health. Further longitudinal research examining personality traits and energy balance–related behaviors across adolescence and into early adulthood will be important for understanding when to intervene.

### Conclusions

This study provides new knowledge about the associations between personality and chronic disease risk factors among young adults. Given the high prevalence of these risk behaviors, their known relationship to important chronic disease outcomes, and the demonstrated effects of personality-targeted programs on other domains of health, the study suggests that personality should be considered when designing preventive interventions for lifestyle risk behaviors, especially high levels of SB, such as screen and sitting time, and low levels of PA. These findings have the potential to inform future development of programs tailored to particular personality styles to improve physical and mental health among young Australians.

## Appendix

Multimedia Appendix 1 Supplementary table.

## Abbreviations

IPAQ-SF: International Physical Activity Questionnaire-Short Form
MVPA: moderate-to-vigorous physical activity
OR: odds ratio
PA: physical activity
RCT: randomized controlled trial
RR: risk ratio
SB: sedentary behavior
SURPS: Substance Use Risk Profile Scale

## Data Availability

The data that support the findings of this study are available on reasonable request from the corresponding author.
