# Supplementary material for: Associations Between Personality Traits and Energy Balance Behaviors in Emerging Adulthood: Cross-Sectional Study
Source: JMIR Public Health Surveill. 2023 Jun 15;9:e42244. doi: 10.2196/42244 (PMC10337402; doi:10.2196/42244)
Supplement: Multimedia Appendix 1 [file publichealth_v9i1e42244_app1.docx]

**Supplementary Table 1:** Associations between personality type and lifestyle risk behaviors

|  | Incidence Rate Ratios or Odds ratios (95% CI) | | | | |
| --- | --- | --- | --- | --- | --- |
|  | **MVPA** | **Sleep Duration** | **Screen time** | **Sitting time** | **Inadequate fruit or veg^a^** |
| **Personality Type** | **RR (95% CI)** | **RR (95% CI)** | **RR (95% CI)** | **RR (95% CI)** | **OR (95% CI)** |
| Hopelessness | **0.93 (0.87-0.99)** | 1.00 (0.98-1.02) | **1.11 (1.08-1.13)** | **1.05 (1.03-1.08)** | 1.17 (0.96-1.42) |
| Anxiety Sensitivity | 0.99 (0.94-1.05) | 1.01 (0.99-1.04) | 1.01 (0.99-1.04) | **1.04 (1.02-1.07)** | 0.86 (0.71-1.03) |
| Impulsivity | **1.16 (1.09-1.23)** | 0.98 (0.96-1.01) | **1.05 (1.02-1.07)** | 0.97 (0.95-1.00) | 1.08 (0.88-1.32) |
| Sensation Seeking | 1.01 (0.95-1.08) | 1.00 (0.97-1.02) | **0.97 (0.94-0.99)** | 1.00 (0.98-1.03) | 0.86 (0.71-1.05) |

^a^Odds ratios derived from logistic regression with reference category as adequate fruit and vegetable intake. All models controlled for sex, employment status, education, and other personality factors. Bold indicates significance at the p<0.05 level.
